# Gut-directed therapy in Parkinson’s disease

**DOI:** 10.3389/fphar.2024.1407925

**Published:** 2024-06-21

**Authors:** Laura Benvenuti, Clelia Di Salvo, Gabriele Bellini, Luisa Seguella, Francesco Rettura, Giuseppe Esposito, Luca Antonioli, Roberto Ceravolo, Nunzia Bernardini, Carolina Pellegrini, Matteo Fornai

**Affiliations:** ^1^ Unit of Pharmacology and Pharmacovigilance, Department of Clinical and Experimental Medicine, University of Pisa, Pisa, Italy; ^2^ Unit of Neurology, Department of Clinical and Experimental Medicine, University of Pisa, Pisa, Italy; ^3^ Department of Physiology and Pharmacology “V.Erspamer”, Sapienza University of Rome, Rome, Italy; ^4^ Unit of Gastroenterology, Department of Translational Research and New Technologies in Medicine and Surgery, University of Pisa, Pisa, Italy; ^5^ Unit of Histology and Medical Embryology, Department of Clinical and Experimental Medicine, University of Pisa, Pisa, Italy

**Keywords:** Parkinson’s disease, microbiota-gut-brain axis, enteric nervous system, enteric inflammation, prebiotics, probiotics, fecal microbiota transplantation

## Abstract

Parkinson’s disease (PD) is a common and slow-progressing neurodegenerative disorder characterized by motor and non-motor symptoms, including gastrointestinal (GI) dysfunctions. Over the last years, the microbiota-gut-brain (MGB) axis is emerging as a bacterial-neuro-immune ascending pathway that contributes to the progression of PD. Indeed, PD patients are characterized by changes in gut microbiota composition, alterations of intestinal epithelial barrier (IEB) and enteric neurogenic/inflammatory responses that, besides determining intestinal disturbances, contribute to brain pathology. In this context, despite the causal relationship between gut dysbiosis, impaired MGB axis and PD remains to be elucidated, emerging evidence shows that MGB axis modulation can represent a suitable therapeutical strategy for the treatment of PD. This review provides an overview of the available knowledge about the beneficial effects of gut-directed therapies, including dietary interventions, prebiotics, probiotics, synbiotics and fecal microbiota transplantation (FMT), in both PD patients and animal models. In this context, particular attention has been devoted to the mechanisms by which the modulation of MGB axis could halt or slow down PD pathology and, most importantly, how these approaches can be included in the clinical practice.

## 1 Introduction

Parkinson’s disease (PD) is a frequent, slow-progressing neurodegenerative disease that affects over six million individuals worldwide with a growing economic impact on society (Ray Dorsey et al., 2018). Common symptoms of PD involve progressive motor dysfunctions, with tremor, bradykinesia, postural instability, and stiffness, caused by the degeneration of dopaminergic (DA) neurons in the *substantia nigra pars compacta* (SNpc) (Kalia and Lang, 2015). Along with the classical PD manifestations (motor symptoms), other symptoms, that can be detected years before central manifestations, include non-motor alterations, such as gastrointestinal (GI) disturbances, rapid eye movement (REM) sleep behavior disorder (RBD), hyposmia, asymmetric nonspecific shoulder pain, depression, and others (Chaudhuri and Schapira, 2009; Gaenslen et al., 2011; Al-Wardat et al., 2024). Nowadays, available therapies might alleviate these symptoms, but does not exist treatment that can halt the disease’s onset and progression (Armstrong and Okun, 2020). Indeed, it is critical to individuate disease-modifying therapies because of PD progression variability and patients’ clinical heterogeneity and so, nowadays, pharmacological treatments are only intended to treat PD symptoms, both motor and non-motor (Jankovic and Tan, 2020). Among the options for the treatment of motor-symptoms, levodopa (L-dopa), the precursor of dopamine, is the most used and effective one almost always combined with carbidopa or benserazide or catechol-O-methyl transferase (COMT) inhibitors, which prevent its peripheral metabolism and increasing its brain bioavailability. Unfortunately, L-dopa showed several side effects, including the worsening of GI symptoms and the appearance of bradykinesia (Djaldetti et al., 1996; Jankovic and Tan, 2020). Monoamine oxidase inhibitors (selegiline, rasagiline), dopamine agonists, anti-cholinergic and anti-glutamatergic are also included in the therapeutic toolkit for PD treatment (Jankovic and Tan, 2020). In the other hand, PD non-motor symptoms are treated with cholinesterase inhibitors (donepezil and rivastigmine) and NMDA receptor antagonists (memantine) that exert beneficial effects in PD-associated dementia and cognitive dysfunctions (Jankovic and Tan, 2020). Unfortunately, many patients with moderate to advanced disease experienced drugs fluctuating response, annoying dyskinesia, and L-dopa-unresponsiveness, which extremely reduce their quality of life (Jankovic and Tan, 2020). In this context, recently device-assisted therapies (DATs), such as deep brain stimulation (DBS) (Okun, 2012), levodopa-carbidopa intestinal gel infusion (LCIG) (Nyholm et al., 2012) and subcutaneous apomorphine injections (Pollak et al., 1989), have been explored and recommended to patients with tremors inadequately controlled by medications. However, research aimed at identifying new therapeutical strategies are imperative to improve quality of life and extend patients lifespan. In the last decade, scientists have pointed out a strict relationship between the brain and the gut microbiota, known as the microbiota-gut-brain (MGB) axis. Indeed, MGB axis alterations are known to affect both intestinal epithelial barrier (IEB) and blood-brain barrier (BBB) integrity, triggering an immune reaction, and potentially increasing brain inflammation and oxidative stress (Tillisch, 2014; Parashar and Udayabanu, 2017; Grillo et al., 2022; Bellini et al., 2023). Recognizing the diverse functions of gut microbiota within the GI tract and the host, we will delve into existing evidence exploring the possibility of targeting the gut microbiota as an innovative avenue for potential disease-modifying therapy in PD. In this review we will provide a comprehensive overview on recent studies suggesting that acting on the gut microenvironment may contribute to ameliorate PD condition, especially focusing on the molecular mechanisms mediated by the several gut-directed therapies and trying to individuate the most appropriate and beneficial one in the different phases of PD.

### 1.1 Pathophysiology of PD

PD is characterized by the aggregation of α-synuclein in Lewy bodies and Lewy neurites in the central nervous system (CNS). Interestingly, similar alterations can be observed in numerous organs, ranging from the skin, salivary glands, and colon, implying that PD could be considered a multiorgan pathology (Chahine et al., 2020). The relationship between early manifestations in the GI tract and later neurodegeneration in the CNS remains unknown. Constipation can appear several years before motor symptoms (Postuma et al., 2019), in accordance with the Braak hypothesis (the veracity of which is debatable) that suppose a primary deposition of α-synuclein within the gastrointestinal system (and in the olfactory bulb) spreading than to the brain through the vagal nerve (Braak et al., 2003a). In this regard, α-synuclein aggregates have been discovered in the dorsal motor nucleus of the vagus nerve (DMV), the locus coeruleus, the olfactory bulb, the submandibular glands, and the enteric nervous system (ENS) in addition to the SNpc (Klingelhoefer and Reichmann, 2015). Misfolded α-synuclein has been reported in enteric neurons of untreated Parkinson’s disease patients even in the early stages of the disease (Cersosimo and Benarroch, 2012). These findings led Braak and his colleagues to suggest that α-synucleinopathy in the ENS might be the earliest PD occurrence, with misfolded α-synuclein spreading to the DMV and then to higher CNS areas via retrograde vagal transmission, contributing to the origins of some cases of PD (Braak et al., 2003b). According to recent research, subdiaphragmatic vagotomy (a surgical method which injures the dorsal and ventral subdiaphragmatic vagus nerve and eliminates vagal afferent (sensory) and efferent (motor) signaling below the diaphragm (Suarez et al., 2018)) can stop misfolded α-synuclein from spreading from the gut to SNpc, which may additionally prevent or delay parkinsonism, confirming the key role of the vagus nerve in the cross talk between the brain and gut (Pan-Montojo et al., 2012; Svensson et al., 2015; Zhang et al., 2020).

Of note, astrocytes and microglial cells could be triggered by misfolded α-synuclein to produce pro-inflammatory cytokines, such as interleukin (IL)-1β and IL-6, contributing to the PD-associated neuroinflammation. Pathological α-synuclein depositions can also cause significant cellular stress, including mitochondrial, lysosomal, and endoplasmic reticulum dysfunctions (Feng et al., 2021). Noteworthy, α-synuclein aggregates could also trigger intestinal and circulating inflammatory/immune cells, leading to the release of pro-inflammatory cytokines, that can infiltrate to the CNS promoting and sustaining the neuroinflammation (De Virgilio et al., 2016; Brown, 2019; Pellegrini et al., 2020). In summary, current research supports a top-down (which begins in the SNpc) and a bottom-up (which begins in the GI tract) origin for PD genesis. Recent research supported this viewpoint by hypothesizing that PD may be separated into two main subtypes: peripheral nervous system (PNS)-first and CNS-first (Miraglia et al., 2015; Horsager et al., 2020). The PNS phenotype, also called body-first, is related with RBD during the prodromal stage and can be recognized by significant autonomic damage as well as digestive symptoms that arise earlier than central dopaminergic system involvement. The CNS-first PD, also known as the brain-first phenotype, is generally RBD-negative during the prodromal phase and is defined by nigrostriatal dopaminergic impairment, which begins without autonomic PNS involvement (Miraglia et al., 2015; Horsager et al., 2020). Despite the remarkable advancement in knowledge relating to the pathophysiology of PD, further investigations are needed to better understand the underlying pathophysiology of the PD subtypes, thus allowing to discover new targets and to develop subtype specific therapies.

### 1.2 The role of the microbiota-gut-brain axis in PD

The GI tract comprises numerous organs that extends from the mouth to the anus and is essential for a variety of activities such as digesting, absorption, and defense against external agents (Yoo and Mazmanian, 2017). In addition, an increasing number of research demonstrates the importance of gut microbiota in preserving brain homeostasis through different mechanisms of interconnection (Powell et al., 2017; Cryan et al., 2019). In particular, MGB communications mostly depend on the gut microbiota’s interactions with the immune system, intestinal barrier, and/or ENS-vagus nerve pathways. Especially, enteric bacteria and their metabolites, such as short chain fatty acids (SCFAs), can directly stimulate enterochromaffin cells to produce various neurotransmitters, such as serotonin, or neuropeptides, such as peptide YY, neuropeptide Y, cholecystokinin, glucagon-like peptide-1 and -2 (GLP-1 and 2), and substance P (Stasi et al., 2017). These substances can then spread into the bloodstream, reach the brain, and directly influence CNS functions (Liu et al., 2020). In addition, specific bacterial products (e.g., SCFAs, vitamins or neurotransmitters, such as acetylcholine, dopamine, noradrenaline, gamma-aminobutyric acid or serotonin) can translocate in the bloodstream through the IEB, reaching the CNS (Matsumoto et al., 2013; Gao et al., 2020; Wenzel et al., 2020). Thus, it seems that neuropeptides, neurotransmitters, and metabolites generated from the circulating microbiota can penetrate the brain and directly impact its neurobiology (Stasi et al., 2012). On the other hand, the CNS alters the intestinal microenvironment by modulating gut motility and neuroendocrine pathways (Gallo and Hooper, 2012; Varatharaj and Galea, 2017; Stasi et al., 2019).

The immune system may be involved in communications across the MGB axis, as intestinal bacteria and their byproducts can activate circulating and intestinal innate and adaptive immune cells. These cells then migrate to the CNS and impact brain functions through the brain lymphatic network (Gorjifard and Goldszmid, 2016). Further research is required as the processes by which immune cells regulate the gut-brain axis are still not widely understood. Notably, it has been discovered that the gut bacteria interact with the ENS-vagus nerve pathways. For instance, altered gut microbiota may stimulate peripheral immunological signals, such as increased levels of pro-inflammatory cytokines (tumor necrosis factor (TNF), IL-6, IL-1, and interferon-γ (IFN- γ)) and reduced anti-inflammatory cytokines (IL-4 and IL-10), which can be detected by the vagus nerve (Cawthon and de La Serre, 2018; Liu et al., 2020).

Changes in the communication processes between the intestinal microbiota and the CNS appear linked to alterations in the composition of the intestinal microbiota. In this regard, variety of research has inquired into shifts in the gut microbiota in PD patients compared to healthy controls (Cirstea et al., 2021; Romano et al., 2021; Toh et al., 2022). According to meta-analyses, the relative abundance of numerous phyla of anti-inflammatory and SCFA-producing bacteria, which includes *Roseburia*, *Lachnospira*, *Blautia*, and *Faecalibacterium*, is lower in PD patients compared to controls. Notably, PD is associated with an increased abundance in opportunistic pathogens and pro-inflammatory bacteria, including *Bacteroides* and *Escherichia* (Toh et al., 2022). *Faecalibacterium* can maintain gut barrier function by producing the SCFA butyrate and secreting anti-inflammatory mediators (Lenoir et al., 2020). Lower levels of butyrate have been associated to postural instability, gait abnormalities, Movement Disorder Society-Sponsored Revised Unified Parkinson’s Disease Rating Scale (MDS-UPDRS) part III motor scores, and depression in PD patients (Li et al., 2017; Xie et al., 2022). Surprisingly, *Lactobacillus*, *Bifidobacterium*, and *Akkermansia* levels are greater in PD patients (Nishiwaki et al., 2020), despite these bacteria are widely recognized as “beneficial”, and often included in probiotic preparations (Tan et al., 2020). The causes for their higher abundance in PD are unknown, although they may be connected to their better adaptation to live in a changed gut environment. Notably, higher *Akkermansia* abundance has been linked to reduced intestinal transit time and low body weight, common features of PD (Fasano et al., 2015; Yong et al., 2020). In PD patients, a decrease in SCFA-producing bacteria and an increase in pro-inflammatory microorganisms is associated with motor and cognitive symptoms (Nishiwaki et al., 2020; Ren et al., 2020) and also with greater fecal calprotectin levels, which is an established clinical biomarker for the evaluation of enteric inflammation (Weis et al., 2019). The increase in *Bacteroides* and *Bifidobacterium* has also been related to increased expression of serum and fecal inflammatory parameters such as IFN-γ or TNF (Lin et al., 2019; Aho et al., 2021). These results highlight the relevant role of gut microbiota in the bidirectional communication between the gut and the brain making itself as a promising target to slow down the progression of PD.

## 2 Therapeutical gut-directed approaches in PD

The involvement of MGB axis in the pathophysiology of PD is pushing the research on the potential therapeutical benefits, in terms of anti-inflammatory and neuroprotective activity, resulting from gut-directed therapies. At present, the most of available studies have investigated the effects of gut microbiota modulation in several experimental models of PD, characterized by similar features to those observed in PD-patients, including GI disturbances (Rota et al., 2019; Pellegrini and Travagli, 2024) and CNS dysfunctions (summarized in Table 1). In addition, several gut-directed therapeutics (Schmidt et al., 2018; Sanders et al., 2019; Zmora et al., 2019) have been found to exert beneficial effects also in PD patients, in terms of improvement of motor and non-motor GI symptoms (Harkins et al., 2020). Accordingly, the following sections and Tables 2, 3 summarize preclinical and clinical data regarding gut-directed therapies in different animal models of PD as well as in patients.

**TABLE 1:** Summary of the main functional, neurochemical, and molecular alterations in GI tract and CNS in different animal models of PD employed to evaluate the effect of gut-directed therapies.

Experimental model	GI Functional alterations	Neurochemical and molecular alterations in GI	CNS Functional alterations	Neurochemical and molecular alterations in CNS	References
Rotenone (mouse)	Faecal retention	No degeneration in ENS	Deficits in long-term objects recognition memory	- Increase in α-syn accumulation in DA neurons	Tasselli et al. (2013), Troshev et al. (2022)
MPTP (mouse)	Delay of colonic transit	- p-α-syn accumulation in colon - Reduction in TH + neurons in ENS - Damage in catecholaminergic neurons in ENS	Deficits in long-term objects recognition memory, memory, and motor coordination	- Reduction in TH + neurons in striatum and DA neurons in SN - Increase in α-syn accumulation	Natale et al. (2010), Mustapha and Taib (2021), Shan et al. (2021)
MitoPark (mouse)	- No differences in gastric emptying and colonic transit time - Faecal ritention	Increase in DA and DOPAC colonic content	Deficits in long-term objects recognition memory, spatial learning and locomotion	- Reduction in DA neurons	Ghaisas et al. (2019), Beckstead and Howell (2021)
A53T α-syn (mouse)	- Slowed colonic propulsion - Increase in colonic dysmotility	p-α-syn accumulation in ENS	Motor and sleeping alterations	- Reduction in DA neurons	Rota et al. (2019), Pellegrini et al. (2022)
Unilateral intranigral injection of 6-OHDA (rat)	- Reduction in peristaltic reflex - Colorectal dysmotility - Faecal retention	- Increase in VIP neurons (*conflicting evidence*) - Reduction in nNOS neurons in distal ileum, proximal colon - Increase in TH e DA transporter in stomach, duodenum and colon - Increase in DA in gastric myenteric plexus - Reduction in D2 expression in proximal and distal colon - No alterations in ChAT neurons in proximal colon (*conflicting evidence*) - Increase in M2R and M3R	Deficits in long-term objects recognition memory	- Reduction in TH + neurons, DA and NA levels in striatum and hippocampus	Colucci et al. (2012), Bonito-Oliva et al. (2014), Fornai et al. (2016b), Pellegrini et al. (2016), Pellegrini et al. (2017), Zhang et al. (2021)

Abbreviations: 6-OHDA, 6-hydroxydopamine; ChAT, choline acetyltransferase; CNS, central nervous system; DA, dopamine; DOPAC, 3,4-Dihydroxyphenylacetic acid; ENS, enteric nervous system; GI, gastrointestinal; M2(3)R, muscarin receptor 2(3); MPTP, 1-methyl-4-phenyl-1, 2,3,6-tetrahydropyridine; NA, noradrenaline; nNOS, neuronal nitric oxide synthase; p-α-syn, phosho-alpha-synuclein; SN, substantia nigra; TH, tyrosine hydroxylase; VIP, vasoactive intestinal peptide; α-syn, alpha-synuclein.

**TABLE 2:** Preclinical studies investigating the effect of gut-directed therapies in different animal model of Parkinson’s disease.

	Model	Treatment	Duration of treatment	Findings	Disease outcomes	References
PROBIOTICS	6-OHDA rats	*Lactiplantibacillus plantarum* PS128	6-week of daily administration (alone or in combination with L-dopa or deep brain stimulation tecnique)	- Rectification of β-oscillations in the primary motor cortex - Reduction of DA cell death - Increment in DA, NA, 5-HT levels in pre-frontal cortex, striatum, and midbrain	Improvements in motor functions	Ma et al. (2023)
		SLAB51: probiotic mixture of *Streptococcus thermophilus, Bifidobacterium longum, Bifidobacterium breve, Bifidobacterium infantis, Lactobacillus acidophilus, Lactobacillus plantarum, Lactobacillus paracasei, Lactobacillus delbrueckii subsp. Bulgaricus* and *Lactobacillus brevi*	*In vivo*: 2 weeks SLAB1 daily administration, 6-OHDA injection and then other 3 weeks of SLAB51 administration	- Increase in TH cells in SN - Reduction of Iba1+ and GFAP + cells - Increase in BDNF, p-TrkB, PPARγ, p-NFR2, NF-kB and HO-1 in SN and striatum - Reduction of apoptotic index for dopaminergic neurons	Improvements in motor functions (i.e., use of controlateral paw)	Castelli et al. (2020)
			*In vitro*: SH-SY5Y cells treated with SLAB51 for 2h and then with 6-OHDA for 24 h	- Activation of neuronal survival pathways (increase in BDNF, p-ERK5, p-TrkB, p-CREB, PI3K, p-AKT, PSD95 levels) - Increase of PPARγ levels - Inhibition of neuronal death pathways (reduction of pro-BDNF, p-JNK, p-ERK, P75 levels) - Reduction of 4-HNE levels	NA	
		*Lacticaseibacillus rhamnosus* HA-114	6-week of daily administration	- NA	Improvements in short-term memory and anxiety-like behaviours	Xie and Prasad (2020)
		Probiotics mixture: *Lactobacillus acidophilus*, *Bifidobacterium bifido*, *Lactobacillus reuteri* and *Lactobacillus fermentum*	14-day treatment with probiotic mixture, starting 7 days before the induction of PD model	- Reduction of MDA levels in the midbrain - Reduction of number of injured neurons (improvements in nuclear pyknosis and neuronal vacuolation)	Improvements in learning and spatial memory and in contralateral rotations behaviour	Alipour Nosrani et al. (2021)
	MPTP-mice	*Bifidobacterium breve* CCFM1067	5-week of daily administration	- Increase in TH + neurons in SN - Reduction of glial reactivity (GFAP and Iba1 levels) in striatum - Reduction of TNF-α, IL-1β and IL-6 levels in striatum and colon - Increase in catalase, GSH, BDNF, GDNF, DOPA, DOPAC, DA and 5-HT levels in striatum - Increase in IL-10 levels in striatum and colon - Increase in ZO-1, occludin, and claudin in striatum and in colon	Improvements in motor functions (i.e., motor agility, balance, coordination)	Li et al. (2022)
PROBIOTICS		*Pediococcus pentosaceus*	4-week of daily administration	- Increase in TH + neurons in SN - Reduction of α-syn levels in SN - Increase in GABA, SOD1, GPx1, Keap1 and Nrf2 levels in brain	Improvements in motor functions	Pan et al. (2022)
		*Lactiplantibacillus plantarum* PS128	4-week of daily administration	- Increase in DA and NA levels - Reduction of DA cell death - Reduction of serum corticosterone levels - Reduction of glial reactivity (GFAP and Iba1 levels) - Increment in BDNF and GDNF levels - Reduction of TNF-α, IL-1β and IL-6 in brain - Increment in SOD, GSH, catalase and GPx in brain	Improvements in motor functions	Liao et al. (2020)
		*Clostridium butyricum*	4-week of daily administration	- Increase in TH + neurons in SN and synapsin I in midbrain - Increase in GLP-1 and GPR41/43 levels in colon - Increase in GLP-1R in the brain - Reduction of CD11b levels in midbrain	Improvements in behavioral deficits	Sun et al. (2021)
	PBMCs from PD patients and healthy controls	*Lactococcus lactis subsp. Cremoris* carrying GLP-1 expression vector	Preventive treatment protocol: daily administration for a total of 2 weeks	- Increase in TH + neurons in SN- Reduction of GFAP, Iba1, α-syn, IL-1β, IL-6 and TNF-α levels in SN	Improvements in exploratory and locomotor functions	Fang et al. (2019)
DIETARY INTERVENTIONS	Rotenone mice	Diet enriched with DHA, EPA and uridine-monophosphate	Diet assumption starts 1 week before the induction of rotenone model	- Increase in TH + neurons in SN - and number of T-cells in colon - Increase in ZO-1 expression in colon and ileum - Increase in colonic length	Improvements in motor functions and coordination; mitigation of GI dysfunctions (increase in intestinal transit time)	Perez-Pardo et al. (2018b)
	A53T α-syn mice	Calcium α-ketoglutarate supplemented diet	3 months of diet assumption before the induction of rotenone model	- Reduction of α-syn and p-α-syn accumulation in SN and striatum - Increase in TH + neurons in SN and striatum - Increase in numbers of synaptic vesicles, VMAT2+ neurons, synapsin and syntaxin levels in SN - Increase in DHA levels in SN - Reduction of IL-1β, IL-6 levels and Iba1+ cells in SN	Improvements in motor functions	Zhang et al. (2023)
	MPTP mice	Fasting mimicking diet 3 days of fasting followed by 4 days of refeeding for three 1-week cycles before the induction of PD model		- Increase in DA and 5-HT levels by reducing neurotransmitters turnover in striatum - Increase in TH + neurons in striatum - Reduction of glial reactivity (GFAP and Iba1 levels) in the SN - Increase in BDNF levels in striatum - Reduction of IL-1β, TNFα levels in striatum	Improvements in motor functions (equilibrium and bradykinesia)	Zhou et al. (2019)
		Ketogenic diet	8-week of daily assumption before the induction of PD model	- Increase in TH + neurons in striatum and SN - Reduction of glial reactivity (GFAP and Iba1 levels) in the SN - Reduction of IL-1β, TNFα, IL-6 levels in plasma, striatum, and colon	Improvements in motor functions	Jiang et al. (2023)
PREBIOTICS	MPTP mice	Sodium butyrate	Oral administration on alternate days for 2 weeks starting 1 week before the induction of PD model	- Reduction of dopaminergic neuronal loss in SN and striatum - Increment in TH + cells and neurons in SN and striatum - Increment in DA, HVA and DOPAC and reduction of DA turnover (DOPAC + HVA)/DA in SN and striatum - Reduction of glial reactivity (GFAP and Iba1 levels) in the SN - Increase in BDNF and GDNF levels in SN	NA	Srivastav et al. (2019)
	Rotenone mice	Diet enriched with prebiotic fibers (GOS, lcFOS, scFOS, nutriose)	Srating from day 28 after the induction of rotenone model until day 93 (65 total day of diet assumption)	- Increment in colonic length	Improvements in motor functions (including GI dysfunctions) and in spatial memory impairments	Perez-Pardo et al. (2018a)
ANTIBIOTICS	MPTP mice	Ceftriaxone	10 days of daily treatment starting 4 days before the induction of PD model	- Increment in TH, GLT-1, BDNF and GDNF in SN - Reduction of α-syn accumulation in SN - Reduction of glial reactivity (GFAP and Iba1 levels) in the SN - Reduction of TLR-4, MyD88 and p-p65 in SN	Improvements in motor functions and exploratory ability	Zhou et al. (2021)
	MitoPark mice (trasngenic mice with *Tfam* gene deletion)	Rifamixin	3 continuative months of rifaximin administered 5 days *per* week	- Reduction of serum concentration of IL-1β, IL-6, TNF-α, claudin-5 and occludin - Reduction of glial reactivity (Iba1 levels) in the SN - Increment in neuronal nuclear protein	Improvements in motor and cognitive functions	Hong et al. (2022)
SYNBIOTICS	MPTP mice	Polymannuronic acid + *Lacticaseibacillus rhamnosus*	5 weeks of daily administration	- Increment in TH + cells and neurons in midbrain and striatum - Increase in BDNF, GDNF, ZO-1 and occludin levels in striatum - Increase in Bcl-2 in striatum	Improvements in motor functions (walking distance and activity)	Liu et al. (2022)
FMT	Rotenone mice	Fresh stool derived from control group mice injected in PD mice	FMT administration daily for 2 weeks starting 4 weeks after rotenone administration	- Increment in TH + cells in SN - Reduction of α-syn accumulation in SN - Reduction of glial reactivity (GFAP and Iba1 levels) in the SN - Improvement in histological score (index of immune infiltration and epithelium disruption) - Increment in ZO-1, occludin and claudin-5 in SN - Increment in ZO-1, occludin and claudin-1 in colon - Reduction of gut permeability (reduction in FD4, LPS and LPB serum concentration) - Reduction of TNF-α, IL-1β and IL-6 serum levels - Reduction of TLR4, MyD88, p-IkB-α, NfKB and increment IkB-α expression in SN and in colon - Reduction of TNF-α, IL-1β, IL-6, iNOS and COX-2 expression in SN and in colon	Improvements in motor symptoms and GI dysfunctions	Zhao et al. (2021)
	MPTP mice	Fresh stool derived from control group mice injected in PD mice	FMT administration daily for 7 days starting 5 days after rotenone model induction	- Increment in DA, DOPAC, HVA, 5-HT and 5-HIAA levels in striatum - Increment in TH + cells and Th expression in SNb- Reduction in glial reactivity (GFAP and Iba1 levels) in the SN - Reduction of TLR4, TBK1, NfKB and TNF-α expression in the colon - Reduction of TLR4, TBK1, NfKB and TNF-α expression in the SN	Improvements in motor symptoms (bradykinesia, muscle strength and equilibrium)	Sun et al. (2018)

Abbreviations: 4-HNE, 4-hydroxynonenal; 5-HT, 5-hydroxytryptamine or serotonin; 6-OHDA, 6-hydroxydopamine; Bcl-2, B-cell lymphoma 2; BDNF, brain-derived neurotrophic factor (p)-ERK5, (phosphorylated)-extracellular signal-regulated kinase 5 (p)-CREB, (phosphorylated)-cAMP, response element-binding protein; DA, DOPA dopamine; DHA, docosahexaenoic acid; DOPAC, 3,4-Dihydroxyphenylacetic acid; EPA, eicosapentaenoic acid; FMT, faecal microbiota transplantation; GABA, gamma-aminobutyric acid; GDNF, glial cell-derived neurotrophic factor; GFAP, glial fibrillary acidic protein (p)-TrKB, (phosphorylated)-tropomyosin receptor kinase B; GI, gastrointestinal; GLP-1, glucagon-like peptide-1; GLP-1R, glucagon-like peptide-1 receptor; GLT-1, glutamate transporter-1; GPR41/43, G-protein-coupled receptor 41/43; GPX1, glutathione peroxidase 1; GSH, glutathione; HO-1, hemeoxygenase-1; HVA, homovanillic acid; Iba1, ionized calcium-binding adapter molecule 1; IL-(1β), (6), (10), interleukin-(1β), (6), (10); KEAP1, Kelch-like ECH-associated protein 1; MDA, malondialdehyde; MPTP, 1-methyl-4-phenyl-1, 2,3,6-tetrahydropyridine; MyD88, Myeloid differentiation primary response 88; NA, noradrenaline; NF-kB, nuclear factor kB; PD, Parkinson’s disease; PI3K, phosphoinositide 3-kinase (p)-AKT, (phosphorylated)-protein kinase B; PPARγ, peroxisome proliferator-activated receptor gamma (p)-NFR2, (phosphorylated)-nuclear factor erythroid 2-related factor 2; PSD95, postsynaptic density protein 95 (p)-JNK, (phosphorylated)-c-Jun N-terminal kinase; SN, substantia nigra; SOD1, superoxide dismutase 1; TH, tyrosine hydroxylase; TLR4, toll-like receptor 4; TNF-α, tumor necrosis factor alpha; VMAT2, vesicular monoamine transporter 2; α-syn, alpha-synuclein.

**TABLE 3:** Clinical studies investigating the effect of gut-directed therapies in PD Patients.

	Subjects	Demographic informations	PD characteristic	Treatment	Duration of treatment	Disease outcomes	References
Dietary interventions	80 PD patients	Age: >40 (mean 58,95) Sex: 41 male, 39 females Race: n.a	1 < H&Y stage ≤5 Average duration of PD (years) in the diet group: 6.6 Average duration of PD (years) in the control group: 5.8	Mediterranean diet	10 weeks	- Amelioration in dimensions of executive function, language, attention, concentration, active memory, and total score of cognitive assessment	Paknahad et al. (2020)
	16 participants: 8 PD patients and 8 controls	Age: 71 (mean) Sex: 8 males, 8 females Race: 15 white, 1 non-hispanic	H&Y stage ≤2.5 in the clinical ‘on’ state	Mediterranean diet	5 weeks	- Significantly lower GSRS scores for constipation and indigestion symptoms - Alterations in intestinal flora	Rusch et al. (2021)
	80 PD patients: 40 mediterranean diet, 40 control diet	Age: 58,95 (mean) Sex 48 males, 32 females Race: n.d	Average duration of PD (years) in the diet group: 6.6 Average duration of PD (years) in the control group: 5.8 Baseline total UPDRS in the diet group: 41.8 Baseline total UPDRS in the control group: 41.5	Mediterranean diet	10 weeks	- Significant reduction of UPDRS. - Significant increase in serum total antioxidant capacity	Paknahad et al. (2022)
	47 PD patients	Age: 40–75 (mean 63) Sex: 31 males, 16 females Race: 44 European, 2 maori, 1 asian	1 < H&Y stage ≤4 in the clinical ‘on’ state	Low-fat or ketogenic diet	8 weeks	- Significant reduction of mds-UPRDS scores, greater in ketogenic group than low-fat group - Amelioration of urinary problems, pain and other sensations, fatigue, daytime sleepiness, and cognitive impairment	Phillips et al. (2018)
	16 PD patients	Age: 36–80 (mean 64.51) Sex: 11 males, 5 females Race: n.a	1 < H&Y stage (mean) ≤4 in the clinical ‘on’ state	Low carbohydrate/healthy fat/ketogenic diet	12 weeks	- Significant improvement in BMI, weight, waist measurement, triglycerides, HgA1C, fasting insulin, HDL, CRP. - Significant improvement in PAS anxiety scale	Tidman et al. (2022)
	25 PD patients	Age: >50 Sex: 13 males, 12 females Race: n.a	H&Y stage (mean): 3.15	Plant-based diet	4 weeks	- Significant reduction of UPDRS total score and motor performances and in H&Y stage	Baroni et al. (2011)
PREBIOTICS	87 participants: 57 PD patients and 30 controls	Age: 40–84 (mean 64) Sex: 43 males, 44 females Race: n.a	Average duration of PD in the RS group and in the dietary instructions (months): 111 Baseline total UPDRS for PD + RS group: 35 Baseline total UPDRS for PD + dietary instructions group: 30	Resistant starch (RS), 5g twice a day orally	8 weeks	- Increase in fecal butyrate - Reduction in fecal calprotectin - Improved non-motor symptoms (NMSQ score) - Reduced depressive symptoms (BDI score)	Becker et al. (2022)
	20 PD patients (including newly diagnosed and treated PD)	Age: mean 64,3 Sex: 11 males, 9 females Race: 20 white/non hispanic	H&Y stage for newly diagnosed and treated PD (mean): 2 Baseline total UPDRS for newly diagnosed: 12 Baseline total UPDRS for treated PD: 14.9	Bar containing resistant starch, rice brain, resistant maltodextrin, and inulin for 10 days (1 bar = 10 g fiber). Once daily for the first 3 days, twice daily for the following week	10 days	- Increase in SCFA-producing bacteria - Reduction of circulating ZO-1 levels and fecal calprotectin levels - Decrease in total UPDRS score	Hall et al. (2023)
PROBIOTICS	40 PD patients: 20 treated with trimebutine, 20 treated with probiotic	Age: mean 76,05 Sex: 17 males, 23 females Race: n.a	H&Y stage (mean): 2.075 Average duration of PD (years): 2.025	Trimebutine 200 mg three times daily or probiotic containing 60mg/tablet of *Lactobacillus acidophilus and Bifidobacterium infantis* twice daily	3 months	- Significant reduction of abdominal pain and bloating - Not significant improvement in constipation	Georgescu et al. (2016)
	60 PD patients: 30 treated and 30 controls (placebo)	Age: 50–90 (mean 67,95) Sex: n.a Race: n.a	Baseline total MDS-UPDRS for the placebo group (0–195): 60 Baseline total MDS-UPDRS for the probiotic group (0–195): 76.2	Probiotic capsule containing *Lactobacillus acidophilus*, *Bifidobacterium bifidum*, *Lactobacillus reuteri*, and *Lactobacillus fermentum* (each 2 × 10^9 ^CFU/g) or placebo	12 weeks	- Decrease in MDS-UPRDS. - Reduction of hs-CRP and MDA levels - Increment in glutathione levels - Significant reduction of insulin levels and insulin resistance - Statistically significant increment in insulin sensitivity	Tamtaji et al. (2019)
	55 PD patients: 27 treated and 28 controls (placebo)	Age: 62–71 (mean 69,75) Sex: 33 males, 22 females Race: 26 Malay, 28 Chinese, 1 Indian	34 PD patients with H&Y < 3 (16 in the probiotic group and 18 in the placebo group) 21 PD patients with H&Y > 3 (11 in the probiotic group and 10 in the placebo group)	*Lactobacillus sp* and *Bifidobacterium sp* (30 × 10^9^ CFU)	8 weeks	- Significant increase in bowel movements - Significant reduction of GTT	Ibrahim et al. (2020)
	72 PD patients: 34 treated and 38 controls (placebo)	Age: mean 69,75 Sex: 41 males, 35 females Race: n.a	Average duration of PD (years) in the probiotic group: 9.7Average duration of PD (years) in the control group: 10.1 Baseline ON-medication MDS-UPDRS part III (0–132) in the probiotic group: 27.9 Baseline ON-medication MDS-UPDRS part III (0–132) in the control group: 27.5	Probiotic capsule containing *Lactobacillus acidophilus, Lactobacillus gasseri, Lactobacillus reuteri, Lactobacillus rhamnosus, Bifidobacterium bifidum, Bifidobacterium longum, Enterococcus faecalis and Enterococcus faecium* (1 × 10^10^ CFU)	4 weeks	- Significant increase in bowel movements - Amelioration in constipation symptoms and stool consistency	Tan et al. (2021)
	46 PD patients: 23 treated and 23 controls 30 non-PD controls	Age: mean 66,5 Sex: 43 males, 44 females Race: n.a	Average duration of PD (years) in the PD-control group: 3.0 Average duration of PD (years) in the PD-probiotics group: 4.5 Baseline UPDRS III in the PD-control group: 19.28 Baseline UPDRS III in the PD-probiotics group: 22.87	*Bacillus licheniformis* (2,5 × 10^9^ CFU, 2 capsules three times daily), *Lactobacillus acidophilus, Bifidobacterium longum, Enterococcus faecalis* (1 × 10^7 ^CFU per strain, 4 capsules/bid)	12 weeks	- Amelioration in bowel movements - Significant reduction of BSS score, PAC-QOL score and degree of defecation effort score - Positive alterations in gut microbiota composition	Du et al. (2022)
	128 PD patients: 63 treated and 63 controls (placebo)	Age: mean 68,43 Sex: 73 males, 55 females Race: n.a	89 PD patients with 1 < H&Y > 3 (46 in the probiotic group and 43 in the placebo group) 39 PD patients with 3 < H&Y > 5 (19 in the probiotic group and 20 in the placebo group)	*Lacticaseibacillus paracasei* strain Shirota fermented milk (100 mL, contain containing 1 × 10^10^ living LcS cells)	12 weeks	- Improvement in constipation-related symptoms (Wexner score, BSFS score, PAC-QOL score, BM) - Amelioration in non-motor symptoms (NMSS, HAMD-17, HAMA)	Yang et al. (2023)
FMT	Case study, 1 PD patient	Age: 71 Sex: male Race: n.a	Years after PD diagnosis: 7 Baseline UPDRS III (ON): 46 Baseline UPDRS II (ON): 13	FMT delivered as 200 mL of solution once *per* day for 3 days *via* transendoscopic enteral tube Donor: 26-year-old healthy male	Follow up at 1 week, 1 month and 3 months	- Improved UPDRS III scores - Resting tremor in legs almost disappeared at 1 week, but slowly reappeared - Gut flora rearrangement, *Firmicutes* increased, *Proteobacteria* and *Bacteroidetes* decreased	Huang et al. (2019)
	15 PD patients	Age: mean 60,66 Sex: 11 males, 4 females Race: n.a	Average duration of PD (years): 4 H&Y stage (mean): 3	Single FMT infusion delivered *via* nasoduodenal tube (n = 5) or colonic delivery (n = 10) Donors: age 18–24 healthy donors	Follow-up between 1 and 12 months	- Decrease in mean UPDRS III scores by month 3 - Improvement in PSQI, HAMD, HAMA, PDQ-39 and NMSQ.	Xue et al. (2020)
	11 PD patients	Age: mean 62,45 Sex: 7 males, 4 females Race: n.a	Average duration of PD (years): 11.8 H&Y stage (mean): 2.27 Baseline UPDRS II: 11.36	Low-carb diet and fasting required before single FMT infusion, delivered *via* nasoduodenal tube Donors: donor bank	Follow-up at 12 weeks	- Improvement in UPDRS, NMSS, PAC-QOL, and Wexner constipation scores - SIBO regression - Specific microbiota abundances fluctuated over time after FMT.	Kuai et al. (2021)
	6 PD patients	Age: mean 63,5 Sex: 3 males, 3 females Race: n.a	Average duration of PD (years): 6.42 H&Y stage (mean): 1.83 PD severity (mild or moderate) 5< Baseline UPDRS III >41	Macrogel bowel preparation used prior to single FMT, delivered *via* colonoscopy Donors: two healthy donors, males aged 38 and 50 years	Follow-up at 2, 4, 8, 12, 16, 20 and 24 weeks	- Significant improvements in UPDRS III, NMSS and Wexner constipation scores	Segal et al. (2021)
	33 PD patients. (SIBO positive)	Age: mean 67.8 Sex: 18 males, 15 females Race: n.a	Average duration of PD (years): 11.7 H&Y stage (mean): 3.6	Rifaximin 200 mg three times a day for 7 days	Follow up at 1 month and 6 months	- Improvement in UPDRS.	Fasano et al. (2013)
ANTIBIOTICS AND BIOLOGICAL DRUGS	Stage 1: 10 PD patients Stage 2: 34 PD patients	Age: 18–86 (mean 60,66) Sex: 11 males, 4 females Race: n.a	10 Patients in Stage 1 Average duration of PD (years): 4.2 H&Y stage (mean): 2.0 Baseline total UPDRS (mean): 53.4 34 Patients in Stage 2 Average duration of PD (years): 6.8 H&Y stage (mean): 2.4 Baseline total UPDRS (mean): 63.2	*Stage 1*: single escalating dose of ENT-01 every 3–7 days beginning at 25 mg and continuing up to 200 mg or the limit of tolerability *Stage 2*: Daily administration of ENT-01, beginning at 75 mg, escalating every 3 days by 25 mg to a dose that had a clear prokinetic effect, or the maximum dose of 250 mg or the tolerability limit. This dose was then maintained (“fixed dose”) for an additional 3–5 days, amounting to at least 7 days on the fixed dose		- Significant improvement in bowel function	Hauser et al. (2019)

Abbreviations: BDI, beck depression inventory; BMI, body mass index; BSFS, bristol stool form scale; BSS, bristol stool scale; CRP, C-reactive protein; FMT, fecal microbiota transplant; GSRS, gastrointestinal symptom-rating scale; GTT, glucose tolerance test; HAMA, hamilton anxiety rating scale; HAMD, hamilton depression rating scale; HDL, high-density lipoprotein; HgA1C, glycated hemoglobin; H&Y, Hoehn and Yahr stage; MDA, malondialdehyde; MDS-UPRDS, Movement disorder society-unified Parkinson’s disease rating scale; NMSQ, Non-Motor Symptoms Questionnaire; NMSS, Non-Motor Symptoms Scale for Parkinson’s Disease; PAC-QOL, patient assessment of constipation quality of life; PAS, panic and agoraphobia scale; PD, Parkinson’s disease; PDQ, pain detect questionnaire; PSQI, pittsburgh sleep quality index; SCFA, short chain fatty acids; SIBO, small intestinal bacterial overgrowth; UPDRS, unified Parkinson’s disease rating scale; ZO-1, zonulin-1.

### 2.1 Preclinical evidence

#### 2.1.1 Antibiotics

Antibiotics are drugs regarded to influence gut microbiota composition by affecting both pathogenetic and beneficial microbes’ population (Fornai et al., 2016a; Becattini et al., 2016). In addition, besides targeting gut microbiota, antibiotics are also endowed with additional properties, such as anti-inflammatory and neuroprotective activities (Stoilova et al., 2013; Sultan et al., 2013; Fornai et al., 2016a; Santa-Cecília et al., 2019). In this context, a previous study by Zhou *et al.* showed how gut microbiota modulation via ceftriaxone, a third-generation cephalosporin active against both Gram-negative and positive bacteria, exerted beneficial effects in 1-Methyl-4-phenyl-1,2,3,6-tetrahydropyridine (MPTP)-induced PD in mice. Authors demonstrated that the administration of ceftriaxone in MPTP mice improved motor dysfunctions and maintained neuronal integrity by restoring brain-derived neurotrophic factor (BDNF) and glial derived neurotrophic factor (GDNF) expression levels in CNS. Such effects were associated with an anti-inflammatory activity, exerted by the downregulation of TLR4 and MyD88, locally restricted to the colon (Zhou et al., 2021). However, more investigations are needed to clarify if such effects depend only on the direct modulation of gut microbiota or also on systemic anti-inflammatory and neuroprotective effects induced by the antibiotic. Notably, a recent and pioneering study has tested antibiotic rifaximin, a poorly absorbed antibiotic with a wide spectrum of antibacterial activity, in PD animals at the early phases of the disease. In particular, Hong and colleagues reported that a 3-month rifaximin treatment protected MitoPark PD mice against neuronal loss and attenuated systemic inflammation, preventing BBB breakdown (Hong et al., 2022). Overall, these findings suggest that a gut-directed therapy aimed at modulating microbiota composition via particular antibiotics can halt disease progression and symptoms, representing a suitable therapeutic option for the management of this neurodegenerative disorder, but more researches are needed to identify the specific activity of any antibiotics and the more effective one.

#### 2.1.2 Dietary interventions

Growing evidence highlights how dietary interventions can influence gut microbiota composition and, in turn, impact on neurological disorders, including PD (Larroya-García et al., 2019). Healthy diet habits have been found to exert neuroprotective effects, decreasing neuroinflammatory responses, oxidative stress, and mitochondrial dysfunctions in the brain (Muth and Park, 2021). Several studies have reported the beneficial effects mediated by different dietary interventions in PD animals. Among all the preclinical models, the rotenone model (which consists in the intravenous, oral, subcutaneous, or intraperitoneal administration of rotenone toxin) represents the most used one because it depicts as fully as possible all the PD features, including GI dysfunctions, olfactory deficits, dopaminergic cell death, neuronal oxidative stress and α-synuclein phosphorylation and aggregation (Radad et al., 2019) (see Table 1 for more detailed information about all the PD preclinical models). Indeed, Perez-Pardo et *al*. investigated the effect of a dietary intervention combining uridine and docosahexaenoic acid (DHA) in rotenone-induced PD mice (Perez-Pardo et al., 2018b). In particular, authors observed that the diet ameliorated motor coordination and exerted a neuroprotective effect, increasing the survival of DA neurons. Moreover, the enriched dietary intervention mitigated GI dysfunctions, α-synuclein accumulation and enhanced zonulin-1 expression in colonic tissues from rotenone-treated mice (Perez-Pardo et al., 2018b). Such effects were attributed both to uridine, a phospholipid precursor, able to increase synaptogenesis and to DHA, regarded to exert anti-inflammatory and antioxidant effects (Perez-Pardo et al., 2018b). Likewise, Zhang and colleagues proved that the dietary intake of α-ketoglutarate ameliorated central α-synuclein pathology, rescued DA neuron degeneration, and suppressed pro-inflammatory mediators such as IL-6, IL-1β and TNF in transgenic A53T α-syn mice via enhancing nigral DHA levels (Zhang et al., 2023). Nevertheless, more studies are needed to better clarify whether the effects resulting from uridine and DHA depend on a gut locally activity or on a direct effect in the brain, since uridine can be transported across cellular membranes in all tissues, including the CNS (Wurtman et al., 2010). Zhou *et al.* showed that a fasting mimicking diet, consisting in 3 days of fasting followed by 4 days of refeeding in MPTP PD mice, reshaped gut microbiota composition and attenuated neuroinflammation by decreasing IL-1β levels and increasing BDNF levels in the striatum, thus offering innovative perspectives for the treatment of PD (Zhou et al., 2019). Similar results were obtained in a very recent study by Jiang et al. in which the administration of a ketogenic diet, characterized by low carbohydrate, high fat, and moderate-protein levels, restructured gut microbiota composition and ameliorated motor dysfunctions in MPTP PD mice (Jiang et al., 2023). Overall, these findings suggest that dietary interventions ameliorate PD and related motor and GI symptoms by exerting anti-inflammatory and neuroprotective effects. However, the exact mechanisms by which such interventions may influence gut microbiota composition, and, in turn, PD progression remain still unclear.

#### 2.1.3 Prebiotics

Prebiotics are primarily indigestible dietary fibers that improve host’s health by selectively promoting the development and/or function of some bacteria in the gut (Davani-Davari et al., 2019). Soybeans, raw oats, unprocessed grains such as barley and wheat, indigestible sugars and oligosaccharides are the most prevalent sources of prebiotics (Markowiak and Ślizewska, 2017). The indigestible fibers, taken with the diet, reach the colon and are locally fermented by several bacteria strains, including *Bifidobacteria* (Hutkins et al., 2016). The final products of the fermentation include SCFAs (butyrate, acetate, and propionate), which promote beneficial activities to the host, such as inhibition of pro-inflammatory genes transcription (i.e., NF-kB, MAPK/ERK) and maintenance of gut barrier function and homeostasis (Blaak et al., 2020). Indeed, Srivastav *et al.* showed that the administration of sodium butyrate counteracted neurotoxicity, mitigated gliosis, and elevated neurotrophic factors levels in brain tissues from MPTP mice, confirming SCFAs as potent neuroprotective agents (Srivastav et al., 2019). Of note, sodium butyrate is in large part absorbed in the upper parts of GI tract and consequently its beneficial effects could be mainly addressed to a direct influence on the brain and, only in a lesser extent, to a gut-directed activity (Pellegrini et al., 2018). On these bases, prebiotic fibers targeting butyrogenic bacteria, such as chitin-glucan and β-glucan, able to promote butyrate production locally in the gut, are emerging for the treatment of PD (Cantu-Jungles et al., 2019). In this context, a recent study showed that the administration of four different monomers of chitosan oligosaccharide protected MPTP mice from DA neuron loss (Wang et al., 2022). Accordingly, several studies in PD mice have shown that non-digestible oligosaccharides such as galacto- and fructo-oligosaccharides (GOS and FOS, respectively) normalized motor and non-motor symptoms, including GI dysfunctions (Perez-Pardo et al., 2018a). The mechanisms underlying GOS and FOS effects resulted from a polarization of microbiota towards beneficial bacteria strain proliferation along with an improvement of synaptic functions in the ENS (Perez-Pardo et al., 2018a). Therefore, GOS and FOS supplementation might confer clinical benefits on PD patients, although more studies are essential to provide further insights on how FOS and GOS beneficially affect PD motor and non-motor symptoms.

#### 2.1.4 Probiotics

Probiotics are live microorganisms able to induce benefits in the host and there are lot of evidence regarding their use in neurodegenerative disorders, including PD (Zommiti et al., 2022). Their beneficial activity results from different mechanisms, such as preservation of barrier integrity through the modulation of tight junctions’ expression (i.e., zonulin-1, occludin and claudin-1) and mucus production as well as reduction of enteric and systemic immune/inflammatory responses (Klaenhammer et al., 2012; Toscano et al., 2017; Sanders et al., 2019). Several efforts have been made to investigate the effects of probiotics and their metabolites in PD animal models, focusing the attention on their effects in halting or slowing down disease progression. Sun *et al.* showed that treatment with *Clostridium butyricum* (Cb) to MPTP mice reshaped gut microbiota composition, augmenting the relative abundance of SCFAs-producing bacteria such as *Akkermansia*, along with a normalization of SCFAs receptor expression, including GPR41/43, as well as an increase of GLP-1 in colonic tissues (Sun et al., 2021). Of note, GLP-1 can cross the BBB and, through the interaction with its receptor (GLP-1R) located with high density on SNpc neurons, astrocytes, and microglia, can, in turn, promote neurogenesis and reverse neuronal loss (Sun et al., 2021). In agreement with the previous results, Fang *et al.* showed how an engineered probiotic, carrying GLP-1 expression vector, ameliorated locomotor impairment, increased tyrosine hydroxylase levels in neurons and counteracted inflammation in MPTP mice, opening the way to implement genetically modified bacteria for the treatment of brain diseases (Fang et al., 2019). Other studies have evaluated the effects of *Bifidobacterium* and *Lactobacillus* in PD animals (Liao et al., 2020; Wang et al., 2022; Li et al., 2022). In particular, Li *et al.* showed that a 5-week supplementation with *Bifidobacterium breve* CCFM1067 suppressed pathogenic bacteria strains (e.g., *Escherichia*-*Shigella*), increased beneficial bacteria growth (e.g., *Bifidobacterium* and *Akkermansia*) and enhanced the expression of tight junction proteins, along with a decrease in pro-inflammatory cytokines levels in colon and brain tissues from MPTP mice (T. Li et al., 2022). Accordingly, a plethora of studies conducted in different animal model of CNS disorders showed that the assumption of probiotics increased intestinal tight junctions’ expression, restored colonic mucus cells loss, that, in turn, can reduce immune/inflammatory responses and alleviate central symptoms (Pellegrini et al., 2023). Another study by Ma and colleagues showed that *Lactiplantibacillus plantarum* PS128, administrated to rats with nigrostriatal dopaminergic neurodegeneration induced by 6-hydroxydopamine (6-OHDA), increased DA content in the striatum and attenuated dopaminergic neuronal loss (Ma et al., 2023), highlighting the neuroprotective effects of *Lactobacillus* supplementation. Interestingly, the neurotrophic effects of *Lactobacillus* and *Bifidobacterium* supplementation were tested in a study conducted by Castelli *et al.* in which SLAB51 (a probiotic mixture of *Streptococcus thermophilus*, *Bifidobacterium longum*, *B. breve*, *Bifidobacterium infantis*, *Lactobacillus acidophilus*, *Lactobacillus plantarum*, *Lactobacillus paracasei*, *Lactobacillus delbrueckii* subsp. *Bulgaricus* and *Lactobacillus brevis*) counteracted dopaminergic neuronal loss and modulated BDNF expression levels in the substantia nigra and in the striatum from 6-OHDA injected mice, by increasing peroxisome proliferator-activated receptor gamma (PPAR-γ) expression levels (Castelli et al., 2020). These findings suggest that the assumption of probiotics containing *Bifidobacterium* and *Lactobacillus* can represent a suitable therapeutical tool to manage PD via decreasing pro-inflammatory responses, counteracting oxidative stress and exerting neuroprotective effects (Magistrelli et al., 2019; Castelli et al., 2020; Xie and Prasad, 2020; Alipour Nosrani et al., 2021). Of interest, some probiotics displayed their potential to release neurotransmitters, including GABA, serotonin, acetylcholine, and noradrenaline that, successively, can influence brain functions. Accordingly, Pan *et al.* showed the ability of *Pediococcus pentosaceus* to counteract α-synuclein accumulation in SNpc, to maintain intracellular redox homeostasis and protect neurons from oxidative damage, through the increment of GABA levels in brain tissues from MPTP mice (Pan et al., 2022). However, whether *P. pentosaceus*-released GABA can influence indirectly brain functions through the maintenance of intestinal homeostasis, or rather it can migrate in the bloodstream and cross the BBB influencing the CNS, remain to be clarified.

#### 2.1.5 Synbiotics

In recent years, researchers are investigating the effects of “synbiotics”, the synergistic combination of pro- and prebiotics, in neurodegenerative disorders (Lye et al., 2018; Ton et al., 2020). In this context, Liu *et al.* tested the administration of *Lacticaseibacillus rhamnosus* GG (LGG) and polymannuronic acid (PM) either alone or in combination (PM + LGG) in MPTP mice (Liu et al., 2022). The results showed that all treatments protect MPTP mice from dopaminergic neuronal loss and neuroinflammation, with a more pronounced beneficial activity in those receiving PM + LGG. Interestingly, the underlying mechanisms of PM, LGG or PM + LGG seem to be different in protecting dopaminergic neurons. In particular, PM displayed an anti-inflammation activity in brain tissues via microbiota derived SCFAs, whereas LGG improved GDNF striatal expressions levels, emphasizing the hypothesis that the synergistic effects of synbiotics are closely dependent on the combination of a pro- and a prebiotic. However, the available preclinical data in PD models about the effectiveness of synbiotics are limited, and more studies are needed to define the most effective combination in slowing down the progression of PD.

#### 2.1.6 Fecal microbiota transplantation (FMT)

Fecal microbiota transplantation (FMT) is a procedure that consists in the transplantation of a gut microbiota extract, obtained from the feces of a healthy donor, into the GI tract of a recipient (Bakken et al., 2013). FMT has been tested in different pathological settings (e.g., *Clostridium difficile* infection, ulcerative colitis, and others) because of its capacity to thoroughly and extensively regenerate gut microbiota, modifying intestinal microbial diversity and restoring atypical intestinal flora (Li et al., 2019; Kim et al., 2021). Several preclinical studies have explored the effect of FMT as a potential therapeutical strategy for the treatment of CNS disorders, including PD. In this regard, Zhao et al. showed that FTM improved both intestinal and motor dysfunctions in rotenone-treated mice and elicited a remarkable reduction of lipopolysaccharide levels in serum, colon, and CNS tissues, underlying how the reshaping of gut microbiota can ameliorate IEB integrity and, successively, reduce immune/inflammatory processes (Zhao et al., 2021). Moreover, based on another study by Sun et *al*., FMT significantly mitigated gut dysbiosis and reduced microglia and astrocyte activation, thus suggesting a significant neuroprotective effect (Sun et al., 2018). Overall, the above findings shed light on the possibility to take advantages through the modulation of MGB axis using FMT practice, although further investigations are necessary to better understand how identify healthy donors, long-term safety, delivery methods, and clinical benefits of FMT.

### 2.2 Clinical evidence

#### 2.2.1 Dietary interventions

Among the numerous dietary regimens, the mediterranean diet (MD) is recognized as a dietary model able to improve health in several disorders, including cardiovascular diseases, cancer, and metabolic syndrome in both Mediterranean and non-Mediterranean populations (Sánchez-Sánchez et al., 2020). In particular, the beneficial effects mediated by the MD seem to be addressed to its content in anti-inflammatory and antioxidant compounds (such as β-carotene and ascorbic acid) and to its ability to control glycemic levels and improve endothelial functions, thus resulting in a normalization of blood pressure levels (Martín-Peláez et al., 2020). Moreover, it is well accepted that a diet intake is responsible for gut microbiota shaping, in terms of composition and function. In this regard, the MD has been observed to positively influence gut microbiota, thanks to its enrichment in dietary fibers and complex carbohydrates which, once fermented by gut microorganisms, lead to the consequent production of SCFAs, capable of regulating glucose and lipid metabolism in several tissues (Martín-Peláez et al., 2020). Recently, a new field of research has showed that a higher adherence to the MD is associated with a lower risk of mental and brain disorders (Aridi et al., 2017). Indeed, case-control studies reported that MD assumption was associated with a decreased risk to develop PD (Alcalay et al., 2012; Maraki et al., 2019). A recent randomized clinical trial examined the impact of the MD on cognitive performance in PD participants. In this study, authors observed that the diet improved cognitive and executive functions, as well as attention, memory, and language (Paknahad et al., 2020). The major source of fat in the MD is olive (*Olea europaea L*.) oil, containing monounsaturated fatty acids, which possesses antioxidant activity and can reduce α-synuclein aggregation (Mohammad-Beigi et al., 2019). Furthermore, the dietary vitamin E and carotene intake from MD were associated with a lower risk of PD, likely minimizing oxidative damage by neutralizing the effect of oxygen free radicals (Yang et al., 2017). Moreover, the ketogenic diet can activate dopaminergic nuclei of the CNS (Naneix et al., 2017) through the increased utilization of ketone bodies by the brain. After 12 weeks, the above effects ameliorate cognitive functions and alleviate both motor and non-motor symptoms in PD patients (Phillips et al., 2018), and improve the feeling of anxiety, described as a persistent condition of worry not connected to stressful events (Tidman et al., 2022). Overall, these findings underscore the potential of tailored nutritional strategies in mitigating the symptoms of PD (Baroni et al., 2011). Further research and understanding of the intricate relationship between diet and PD may pave the way for innovative approaches in the management of this neurodegenerative disorder.

#### 2.2.2 Prebiotics

The use of prebiotics in PD patients has been demonstrated to improve GI peristalsis, enhance immunological function, and alleviate constipation. In one small open-label investigation, a diet high in insoluble fibers (a typical prebiotic food source) ameliorated constipation, motor capacities and systemic L-dopa bioavailability (Astarloa et al., 1992). Furthermore, in an open-label trial enrolling 87 participants, the authors found that individuals with PD receiving resistant starch improved non-motor symptom ratings and had also substantially incremented fecal butyrate levels, as well as decreased fecal calprotectin levels, compared to patients that received dietary recommendations only (Becker et al., 2022). In another open-label study, the administration of a prebiotic mixture to PD patients determined the decrease in total UPRDS score and a reduction in both intestinal inflammation and circulating zonulin levels, a marker of IEB dysfunction (Hall et al., 2023). Therefore, prebiotics could have beneficial effect on PD by stimulating the production of SCFA-producing bacteria and their metabolites. However, there is currently scarce clinical data on prebiotics in PD patients, hence more trials are needed to adequately study the process.

#### 2.2.3 Probiotics

Many studies evaluated the effect of multistrain probiotics in the treatment of PD constipation (Georgescu et al., 2016; Ibrahim et al., 2020; Tan et al., 2021; Du et al. 2022). Tan and colleagues observed a significant increase in bowel movements and constipation symptoms in PD patients treated with probiotic capsules containing strains of *Lactobacillus*, *Bifidobacterium*, and *Enterococcus* (Tan et al., 2021). In another study with a different formulation containing *Lactobacillus*, *Bifidobacterium*, and *Enterococcus* the researchers also found a significant reduction in Bristol stool scale (BSS) score, patient assessment of constipation quality of life questionnaire (PAC -QOL) score and degree of defecation effort score, associated with positive changes in the gut microbiota composition of PD patients (*n* = 46) (Du et al., 2022). Other studies have evaluated the effect of probiotics in motor and non-motor symptoms (Tamtaji et al., 2019; Yang et al., 2023). Tamtaji et al. in their randomized, double-blind trial (*n* = 60) observed that a 12-week treatment with probiotic capsules containing *L. acidophilus*, *Bifidobacterium bifidum*, *Lactobacillus reuteri*, and *Lactobacillus fermentum* determined a significant decrease in MDS-UPRDS, other than reducing inflammatory parameters and oxidative stress. In a recent research, involving 128 PD patients, a fermented milk containing *Lacticaseibacillus paracasei* strain Shirota was able to improve constipation-related symptoms but also ameliorate other non-motor symptoms (i.e., depression and anxiety) (Yang et al., 2023). Overall, probiotics are likely to represent a promising therapeutic strategy for the treatment of PD-related GI symptoms, but their safety needs to be further evaluated, as *Enterococcus* spp., often incorporated in probiotics, has shown significant levodopa breakdown capability (Rekdal et al., 2019), stressing the need for caution during its utilization.

#### 2.2.4 Fecal microbiota transplantation

Considering the association between GI symptoms and PD, FMT might represent a possible therapeutical strategy. Several trials (NCT04854291, NCT05204641 and NCT04837313 on ClinicalTrials.gov) are at present being conducted to explore the impact of FMT on both motor and non-motor symptoms in PD. In 2019, the first human case report including the employment of FMT in PD was published (Huang et al., 2019). FMT was performed in a 71-year-old man who had a 7-year history of PD. The patient got nearly immediate alleviation from constipation, and his tremor in his lower extremities was also significantly decreased and remained reduced at the 3-month follow-up (Huang et al., 2019). In a more recent study, Segal and colleagues examined a group of six PD patients who received FMT (Segal et al., 2021). Four weeks after FMT, most of patients showed improvements in their PD-related constipation and an amelioration in UPDRS-III and Non-Motor Symptoms Scale scores, with few adverse effects reported (Segal et al., 2021). FMT from healthy donors, administered via a nasoduodenal tube, has also demonstrated to elicit significant benefits for both motor and non-motor PD symptoms (Kuai et al., 2021). In another study conducted on 15 patients, at a 1-month follow-up, it was observed that FMT was linked with better sleep and overall quality of life, in addition to decreases in motor symptoms, anxiety, and depression scores (Xue et al., 2020). A 3-month follow-up of the same population revealed long-term gains among the 12 participants who remained in the trial. Interestingly, ten of the fifteen patients in the trial received colonic FMT, while the remaining five received FMT through a nasoduodenal tube. The latter group showed major improvements on several motor and non-motor PD-related evaluations, as compared with patients receiving FMT via the colonic method, indicating that method of delivery may be a significant variable in FMT success (Xue et al., 2020). The adverse effects observed in the previous studies were minor to mild and of short duration (Huang et al., 2019; Xue et al., 2020; Kuai et al., 2021; Segal et al., 2021). Overall, this research shows that FMT could represent a feasible treatment for PD. Still, given the different rates of response in relation to FMT delivery, more research is needed to determine the appropriate route of administration, the choice of proper endpoints, and the rate of administration required to sustain desired beneficial effects.

#### 2.2.5 Postbiotics, antibiotics, small molecules and biological drugs

Among the antibiotics, rifaximin, has been used to treat small intestinal bacterial overgrowth (SIBO) also in PD. In a recent study, involving 33 PD patients, SIBO eradication with rifaximin resulted in lower GI motor fluctuations, but without ameliorating L-DOPA bioavailability (Fasano et al., 2013). Additional microbial-directed therapies having the potential to assist PD patients, such as postbiotics, small-molecules and biological drugs, have yet to be fully investigated in the clinical context. SCFAs administration and selective blocking of levodopa-metabolizing bacterial enzymes appear to offer special promises, although more testing in human interventional research is required. Other immunomodulatory and IEB-restoring therapies need more consideration. Anti-TNF treatment, which has been linked to a lower PD incidence in IBD patients (Peter et al., 2018), has been shown to change fecal microbiota composition, with fecal amounts of butyrate and SCFAs in general being linked to clinical remission (Aden et al., 2019). Another new frontiers could be targeting PD-related molecular pathogenesis (for example, involving α-synuclein or glucocerebrosidase) in the ENS. In an open label trial, orally administered squalamine (that seems to relocate α-synuclein aggregates from ENS neurons) was safe and significantly reduced the symptoms of constipation in patients with PD (Hauser et al., 2019).

## 3 Future challenges and perspectives

Despite the promising results of gut-directed therapies in PD, certainly, several questions remain open. For instance, the introduction of probiotics in the clinical practice (i.e., combo-therapy bacteria-drugs) needs furthers investigations. Indeed, some bacteria species, endowed with a specific enzymatic activity, such as *Enterococcus faecalis, Lactobacillus fermentum* and *Lactobacillus plantarum* were found to lower L-dopa bioavailability, converting L-dopa in dopamine in the intestine, even in the presence of human peripheral decarboxylase inhibitors (i.e., carbidopa). Such an activity reduces L-dopa brain levels, thus decreasing drug beneficial outcomes (Cirstea et al., 2023). In parallel, L-dopa treated patients showed an increment in *Peptoniphilus*, *Finegoldia* and *Enterococcus* relative abundance, along with a decrease in *Faecalibacterium*, *Blautia* and *Lachnospirae* relative abundance in comparison with L-dopa-untreated-PD patients, demonstrating that L-dopa, itself can influence gut microbiota composition (Weis et al., 2019). Accordingly, L-dopa assumption induces gut dysbiosis, that, in turn, can contribute to GI symptoms observed in L-dopa treated patients (Chen et al., 2021). Likewise, the utilization of DATs for the treatment of long-term PD has a notable impact on gut bacteria species (Melis et al., 2021; Lubomski et al., 2022), which were found altered following DATs initiation in comparison with PD-control patients, but clinical trials are scarce and further investigations are necessary in this field (Hey et al., 2023). However, it is necessary to better clarify the pharmacokinetic interactions between PD drugs and gut-directed therapies and individuate the better combo bacteria-drugs regimen with the aim to creating a boosted beneficial impact on PD symptoms and progression. Indeed, certainly, *Lactobacillus* can reduce L-dopa bioavailability, but at the same time have demonstrated the capacity to slow down PD progression and counteract PD symptoms. Therefore, *Lactobacillus* could represent a suitable gut-directed therapy in the pre-motor phases of PD before the beginning of L-DOPA treatment. In addition, other limitations regard the difference therapy response in male and female, since gut microbiota is influenced (and, in turn, can influence) by sex hormone levels, especially estrogens (Rizzetto et al., 2018). In this context, further clinical studies should be performed in sex-oriented groups.

## 4 Conclusion

**FIGURE 1:**
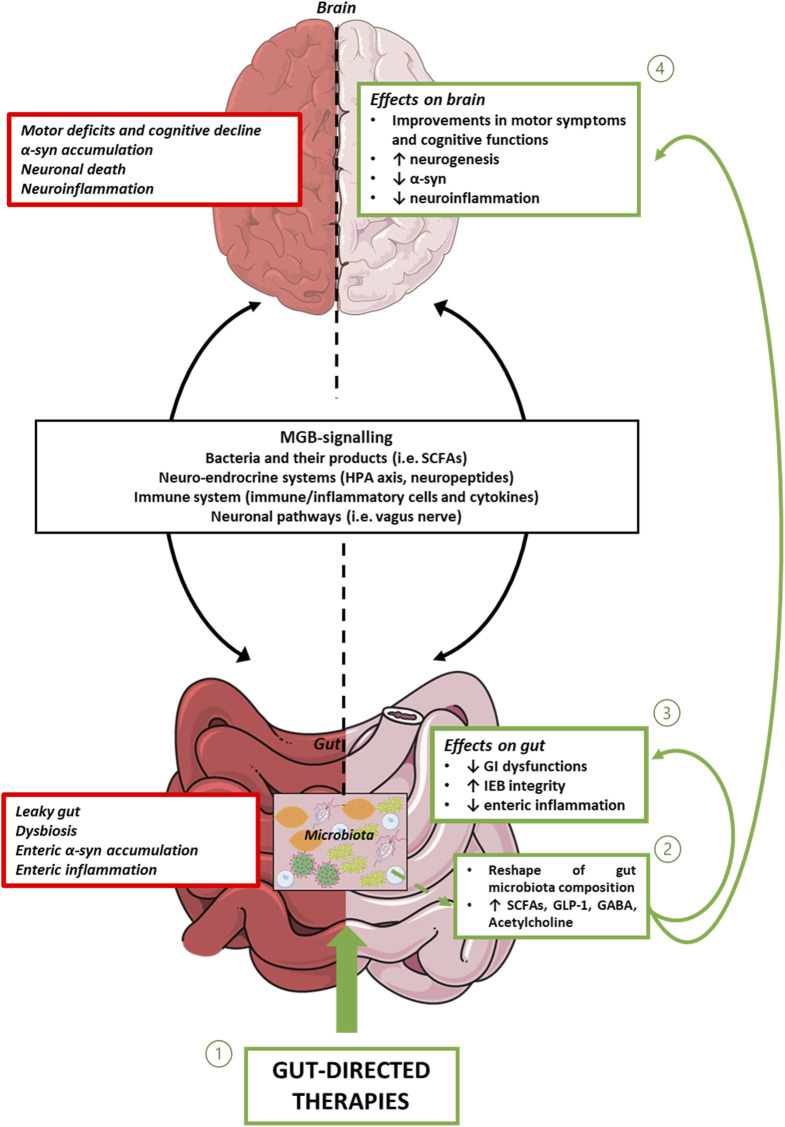
Representative diagram showing MGB signalling, and intestinal and brain alterations associated with PD. In this context, gut-directed therapies (1), through the reshaping of gut microbiota composition and the consequent release of beneficial products, including bacterial metabolites and neurotransmitters (2), besides counteracting enteric inflammation, reinforcing gut barrier integrity, and improving intestinal symptoms (3), exert beneficial effects on CNS alterations. Abbreviations: α-syn: α-synuclein; CNS: central nervous system; GABA: γ-Aminobutyric acid; GI: gastrointestinal; GLP-1: glucagon-like peptide-1; IEB: intestinal epithelial barrier; HPA axis: hypothalamic–pituitary–adrenal axis; MGB: microbiota gut-brain axis; PD: Parkinson’s disease; SCFAs: Short-chain fatty acids.

**FIGURE 2:**
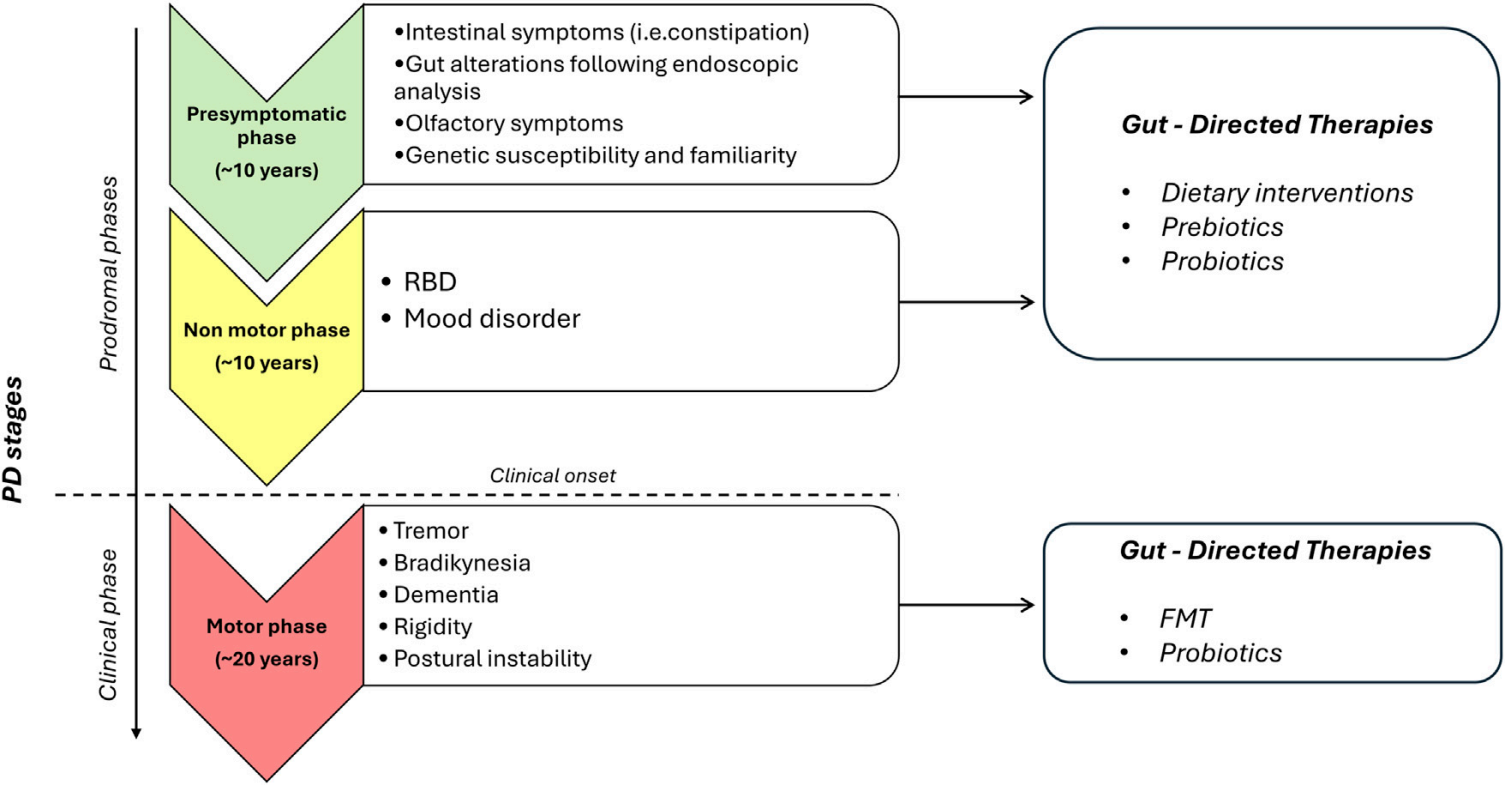
Proposed diagram for the potential application of gut-directed therapies based on PD stages. The present diagram is based on clinical and preclinical studies, so it should always be interpreted in conjunction with patient clinical history, clinical examinations, and PD drugs assumption. For patients in the prodromal stages of PD with intestinal symptoms and gut alterations following endoscopic analysis and subjects with RBD and/or mood disorder, gut-directed therapies, including dietary interventions, prebiotics, and probiotics, can be proposed. For patients in the clinical phase of PD, additional gut directed therapies, including FMT and probiotics, can be added to validated PD drugs. Abbreviations: PD, Parkinson’s disease; RBD, rapid eye movement (REM) sleep behavior disorder.

In conclusion, the present review provide evidence about gut-directed microbial therapies in the context of PD and how they represent a promising frontier in our understanding and potential treatment of this complex neurodegenerative disorders. The intricate interplay between the gut microbiota and the CNS is increasingly recognized as a significant factor in the development and progression of PD. While the precise mechanisms underlying these interactions are still being elucidated, the potential therapeutic implications are substantial. Still, a lot of questions remain regarding how MGB influences the onset and the progression of PD and the potential involvement of ENS together with how to identify the optimal probiotics strains to employ, the choice of one-single strain or multi-strains, the inter-subject’s variability, the appropriate administration schedule, the safety of probiotics and the lasting of their effects. Furthermore, for FMT, while case studies are useful as inspiration for future research, at present clinical trials are limited and lack in standardization (Seguella et al., 2023). However, the concept of modulating the gut microbiota through microbial therapies, such as probiotics, prebiotics, or FMT, holds promise as a novel approach to manage or even mitigate the symptoms of PD (Figure 1). These therapies have the potential to restore microbial balance, enhance the production of beneficial metabolites and exert a positive influence on immune function, thereby offering hope for beneficial outcomes not only in PD, but also in other brain disorders characterized by intestinal symptoms, gut dysbiosis, enteric barrier alterations and activation of inflammatory pathways such as Alzheimer’s disease, depression, and autism (Pellegrini et al., 2018). However, several limitations in the translational from preclinical model to clinical practice are still present. For instance, preclinical models, though reflect the main neuropathological and clinical features of PD, do not completely reproduce the pathophysiology seen in humans. Indeed, environmental factors and genetic susceptibility play a role in the onset and progression of PD in humans. Moreover, it is fundamental to set up specific studies on PD patients selected on the disease phase to better identify the most effective timing for gut-directed therapies administration and their integration with the commonly used pharmacological treatments (in Figure 2, a diagram for the potential application of gut-directed therapies based on PD stages is proposed). Based on the results discussed in the present review, we can speculate that the gut-directed therapies could be used both as disease-modifying therapies as well as symptomatic therapies. Indeed, they have displayed their capacity to reduce PD symptoms (i.e., motor and intestinal symptoms) in several clinical studies, but only preclinical evidence is available about their effect in reducing dopaminergic cell death or decreasing the accumulation of α-syn. In this context, more clinical studies are imperative to confirm the above evidence also in PD patients. However, it is essential to acknowledge that further research is needed to fully understand the complexities of this relationship, to develop safe and effective therapies tailored to unique needs of PD patients. In summary, the emerging field of gut-directed microbial therapies in PD holds significant promise for enhancing our therapeutic arsenal against this debilitating condition. While challenges and questions remain, the potential benefits for patients and their quality of life make this an area of continued exploration and innovation in the quest to conquer PD.
